# A mark-resight survey method to estimate the roaming dog population in three cities in Rajasthan, India

**DOI:** 10.1186/1746-6148-7-46

**Published:** 2011-08-11

**Authors:** Lex R Hiby, John F Reece, Rachel Wright, Rajan Jaisinghani, Baldev Singh, Elly F Hiby

**Affiliations:** 1 Conservation Research Ltd., 110 Hinton Way, Great Shelford, Cambridge CB22 5AL, UK; 2 Help in Suffering, Maharani Farm, Durgapura, Jaipur 302018, Rajasthan, India; 3 Tree of Life for Animals, Kharekhari Village, Nr Foy Sagar, Ajmer 301005, Rajasthan, India; 4 Marwar Animal Protection Trust, Maanpura, Dardyaon ki Dhani, Jhalamand - Khejarli Road, Jodhpur, Rajasthan, India; 5 World Society for Protection of Animals, 222 Grays Inn Road, London, WC1X 8HB, UK

## Abstract

**1. Abstract:**

**Background**

Dog population management is required in many locations to minimise the risks dog populations may pose to human health and to alleviate animal welfare problems. In many cities in India, Animal Birth Control (ABC) projects have been adopted to provide population management. Measuring the impact of such projects requires assessment of dog population size among other relevant indicators.

**Methods**

This paper describes a simple mark-resight survey methodology that can be used with little investment of resources to monitor the number of roaming dogs in areas that are currently subject to ABC, provided the numbers, dates and locations of the dogs released following the intervention are reliably recorded. We illustrate the method by estimating roaming dog numbers in three cities in Rajasthan, India: Jaipur, Jodhpur and Jaisalmer. In each city the dog populations were either currently subject to ABC or had been very recently subject to such an intervention and hence a known number of dogs had been permanently marked with an ear-notch to identify them as having been operated. We conducted street surveys to record the current percentage of dogs in each city that are ear-notched and used an estimate for the annual survival of ear-notched dogs to calculate the current size of each marked population.

**Results**

Dividing the size of the marked population by the fraction of the dogs that are ear-notched we estimated the number of roaming dogs to be 36,580 in Jaipur, 24,853 in Jodhpur and 2,962 in Jaisalmer.

**Conclusions**

The mark-resight survey methodology described here is a simple way of providing population estimates for cities with current or recent ABC programmes that include visible marking of dogs. Repeating such surveys on a regular basis will further allow for evaluation of ABC programme impact on population size and reproduction in the remaining unsterilised dog population.

## 2. Background

With the spread of urbanisation throughout the developing world the population of roaming dogs (i.e. dogs that are neither confined nor restricted, sometimes known as "street", "stray" or "free-ranging" dogs) in urban areas has the potential to increase. Such dogs can suffer from welfare problems and can present a human health risk, most notably rabies, with 99% of human rabies deaths due to rabies transmission from infected dogs [1].

Human mortality from endemic canine rabies has been estimated to be 55,000 deaths per year globally with 19,713 occurring in India [2]. Animal Birth Control (ABC) has been adopted in many Indian cities through sterilisation and vaccination of captured roaming dogs and their release back into their original territories in order to reduce the risk of rabies transmission, stabilise the free roaming dog population and potentially reduce its size. This approach is also outlined in Indian legislation under the Animal Birth Control (Dogs) Rules, 2001.

Opinions are divided as to the effectiveness of such programmes in controlling the number of roaming dogs (e.g. [3] and [4]) yet the data needed to assess and optimise their effectiveness are largely lacking. Population surveys may provide information relating to population size and demography that, alongside data relating to rabies incidence, can support evaluation of ABC programme impact.

**Figure 1:**
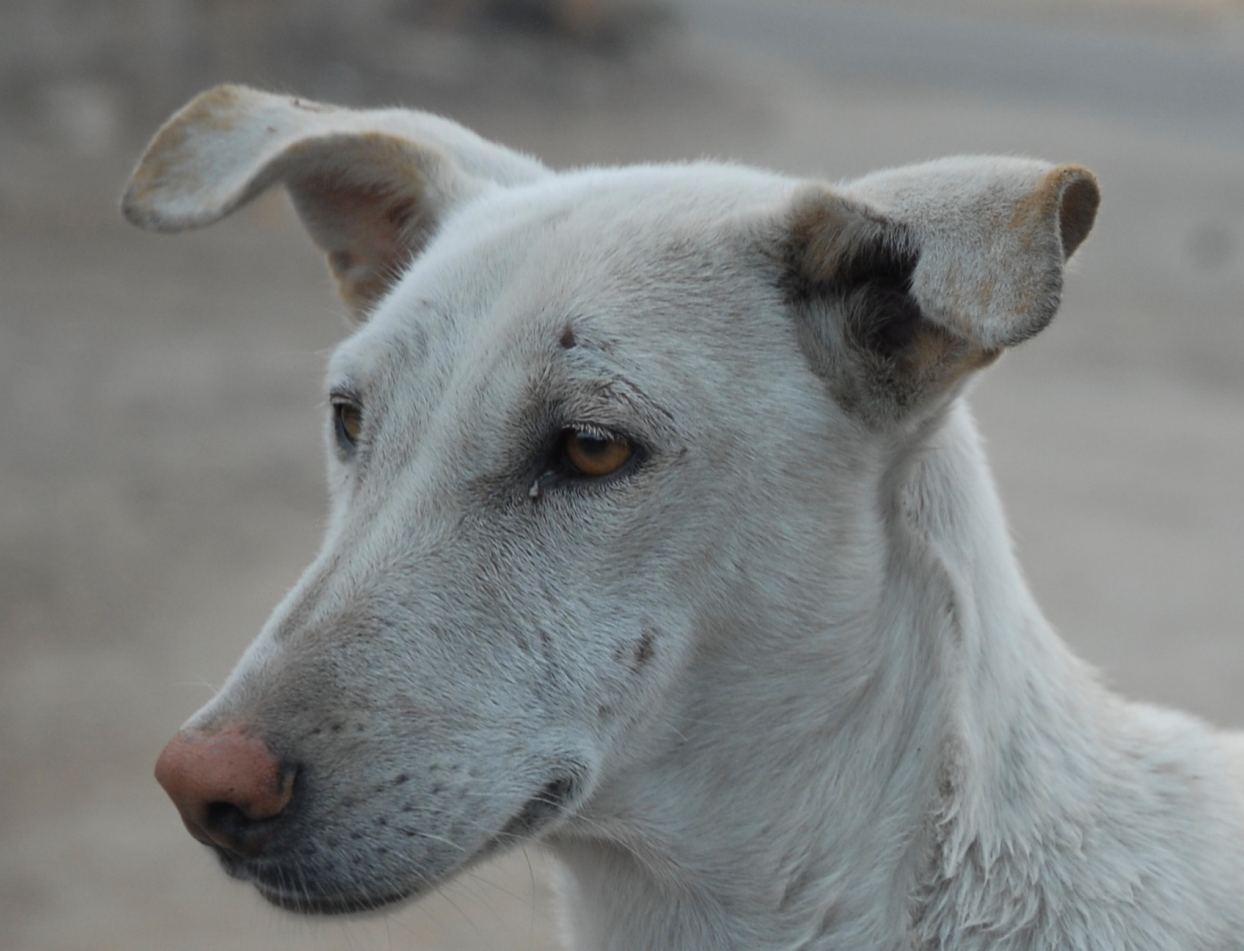
**Photo of dog in Jaisalmer with ear-notch visible on leading edge of left ear**. - All dogs subject to sterilisation and/or vaccination as part ABC projects are ear-notched using a clamp and cauterizer whilst under anaesthetic. An ear-notch provides a permanent and visible mark that the dog is already sterilised and hence avoids them being caught unnecessarily. This photo shows a dog in Jaisalmer with an ear-notch on the leading edge of the left ear.

This paper describes a mark-resight survey methodology used in three cities in Rajasthan, India (Jaipur, Jodhpur and Jaisalmer). The roaming dog populations in all three cities were either currently subject to an ABC programme or had been very recently subject to such an intervention. The ABC programme in Jaipur is run by Help in Suffering (HIS) in collaboration with the Jaipur Municipal Council and has spayed and vaccinated 70,000 dogs between 1995 and the end of 2009. The ABC programme in Jodhpur is run by the Marwar Animal Protection Trust (MAPT). The programme started in 2004, up until November 2009 a total of 23,723 females and 25,037 males had been sterilised and vaccinated (for further details see [5], which also considers change in the Jodhpur roaming dog population following the intervention). In May and June 2009, the Tree Of Life For Animals (TOLFA) caught 1,000 male and female dogs for sterilisation and vaccination in Jaisalmer. At the time of sterilisation and vaccination (or vaccination only in the case of adult males in Jaipur) all dogs are ear-notched whilst under anaesthetic before being released at the point of capture. It is this ear-notch 'mark' that is then used during the resight stage of the survey. Re-catching is not required as these notches are easily visible on the leading edge of the ear (Figure 1). Resight data was collected using two methods; either during specific surveys to estimate the percentage of ear-notched dogs or opportunistically at the time of capturing dogs for ABC when all dogs observed are required to be checked for the presence of an ear-notch.

Methods for estimating numbers of roaming dogs include questionnaire surveys ([4] and [6]), mark-resight using paint sprays [5], distance methods [7] and exhaustive counts of randomly selected city blocks [8]. Each of these methods has the potential to make an initial estimate of the size of the roaming dog population as required for planning of an intervention. However as a way of monitoring its effects over a large area these methods may need too great an investment of time and resources. We suggest that reliable population monitoring can be achieved with limited resources by exploiting the existence of the large number of marked individuals accumulating as a result of the intervention itself. For example, a previous study [9] was able to estimate the dog population of N'Djamena in Chad using household and street surveys to record the percentage of dogs with collars that had been applied during a mass vaccination campaign a few days before. In this paper we extend this idea to dogs ear-notched during an ABC programme over much longer periods by allowing for an estimated rate of mortality in these dogs.

We use model M_t _[10] to derive the estimator. Thus in common with many other mark-resight surveys we assume that, although sighting probabilities may vary over time, they are equal for marked and unmarked roaming dogs, the marks are not lost, and the population is closed with respect to immigration and emigration. In contrast to other mark-resight methods we do not need to assume equal rates of mortality for marked and unmarked dogs because our estimate of the mortality of marked dogs is used explicitly to estimate the size of the surviving marked population.

## 3. Results

### Population estimates

#### Jaipur

**Figure 2:**
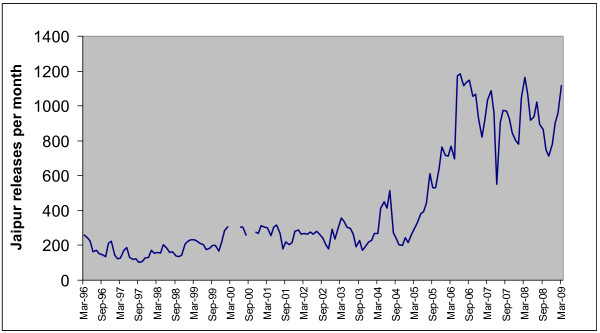
**Total monthly releases of ear-notched dogs in Jaipur since intervention began**. - In Jaipur, since the ABC project commenced, a number of dogs have been released every month after sterilisation and vaccination, or vaccination only in the case of adult males. Each of these dogs was ear-notched whilst under anaesthetic before release. This figure shows the monthly total of ear-notched dogs released every month by the project.

**Figure 3:**
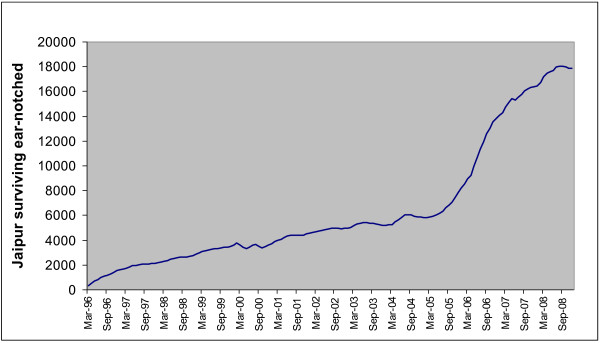
**Estimated number of surviving ear-notched dogs each month since the intervention began using an annual survival of 0.70**. - Each ear-notched dog was assumed to have an equal chance of survival following release. An annual survival estimate of 0.7 was used from a previous study also conducted in Jaipur [11]. Hence for each month since the ABC project started the number of ear-notched dogs estimated to still be alive at that time was calculated and is shown in the graph.

Figure 2 illustrates total monthly releases since the start of the intervention in Jaipur and Figure 3 the estimated number of surviving ear-notched dogs using an annual survival of 0.70. The increase since 2005 is the result of collecting males at any age for vaccination, whereas prior to 2000 males were not collected and prior to 2005 only young males were collected for vaccination and castration. The city is divided into ten zones and the number of zones included in the intervention has increased over time. Therefore the number of surviving ear-notched dogs was estimated separately for each zone and combined with the observed percentage of ear-notched dogs in each zone to give the total estimate of 36,580 roaming dogs in the whole city in November 2009. Based on variation in percentage ear-notched over the ten zones the coefficient of variation (CV) for the average percentage was estimated at 0.043 and combined with a CV of 0.13 for the estimate of annual survival gives 95% confidence limits from 26,562 to 46,597 roaming dogs.

The estimates given are based on the observed percentages of ear-notched dogs whereas the survival estimate of 0.70 is for spayed females [11]. However, recalculating the estimates based on observed percentages of ear-notched females makes little difference; the latest estimates are within 1% of each other.

**Figure 4:**
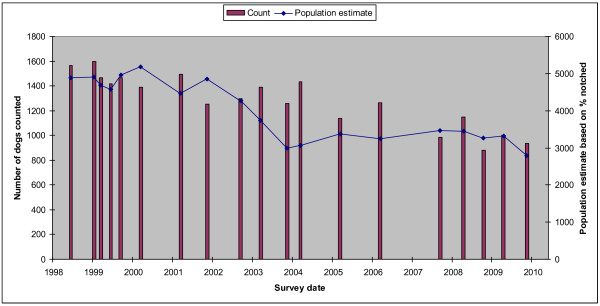
**Comparison between counts of dogs and population estimates based on percentage of ear-notched dogs**. - Surveys have been conducted in the Pink City region of Jaipur since 1997. The data from these surveys can be used to compare the number of dogs counted during street surveys in the Pink City region to the population estimate based on the percentage of ear-notched dogs observed and the estimated number of surviving ear-notched dogs at the time of observation. This graph compares the trajectory the Pink City region counts themselves to the Pink City region female roaming dog population estimates based on observed percentage ear-notched.

In 9 of the 10 zones the observed percentages of ear-notched dogs were based on records that have been collected opportunistically by the catching teams since February 2007. However in the Pink City zone biannual street counts have been conducted since February 1997. Figure 4 compares the trajectory of Pink City female roaming dog population estimates based on observed percentage ear-notched in those counts to the Pink City counts themselves.

#### Jodhpur

Up until November 2009, the ABC programme in Jodhpur had marked and released 48,760 dogs.

Of the 477 dogs observed during the surveys 78% were ear-notched, almost identical to the 77% observed by the catching team supervisors during the catching operations. Using the estimate of 0.7 for annual survival the number of marked dogs estimated to be alive in the city was 19,137 representing 77% of the total number of roaming dogs, giving a point estimate of 24,853 roaming dogs. The CV for the percentage ear-notched was 0.029 (based on the variance of that percentage over the different areas of the city). In combination with the CV of 0.13 for annual survival, 95% confidence limits for the total roaming dog population of Jodhpur in November 2009 are 18,364 to 31,341.

#### Jaisalmer

**Figure 5:**
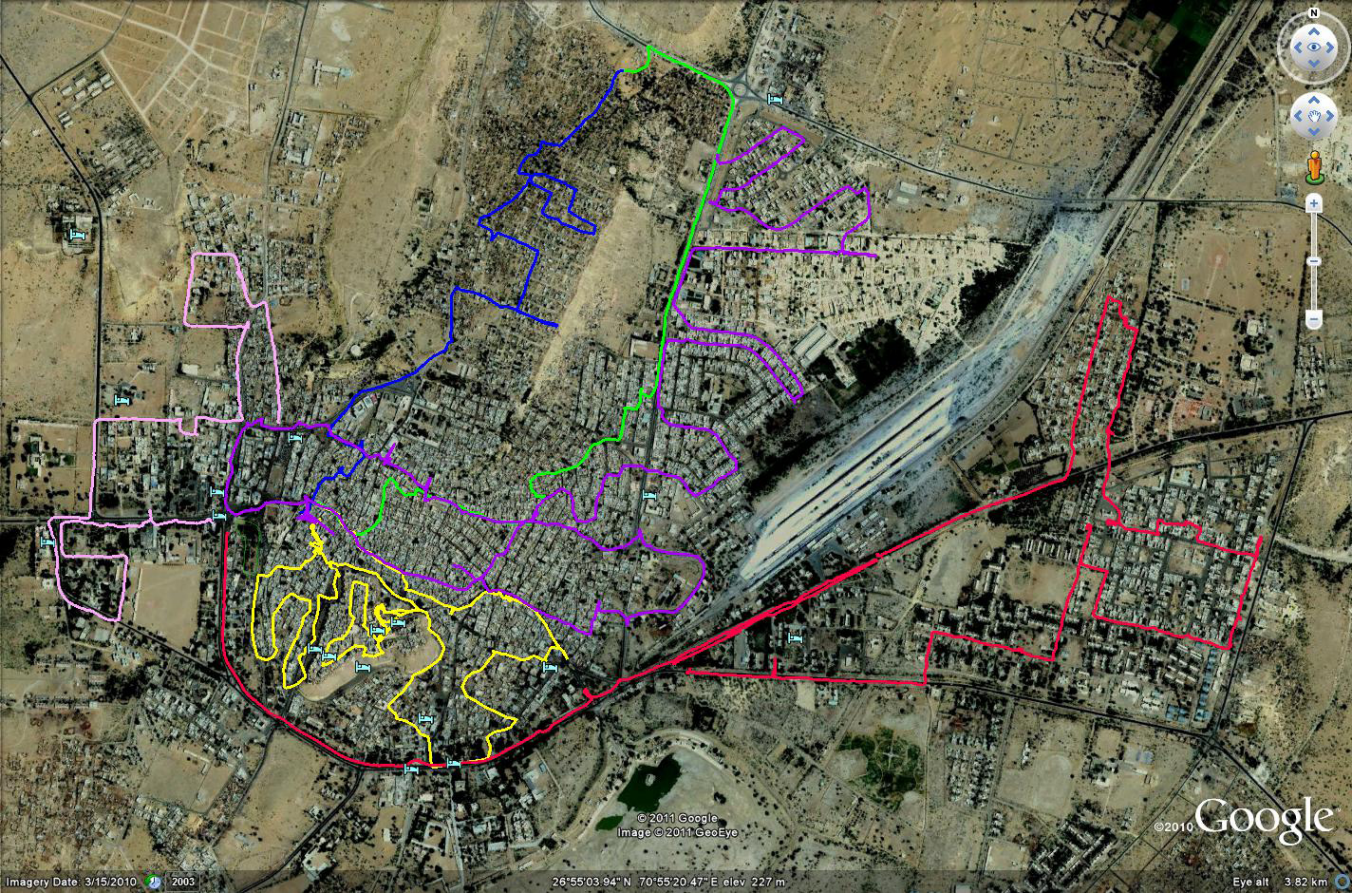
**Google image with coloured lines showing the tracks walked or cycled along streets in Jaisalmer during survey**. - Detailed street maps of Jaisalmer were unobtainable hence an aerial map from the Google Earth website (http://earth.google.co.uk/) was downloaded to a hand-held microcomputer running the GPS*GO *mapping program under Windows 6 (Craporola Software Inc). A GPS receiver was then used to mark a track on the map as the survey team walked or cycled along the streets to ensure a roughly even coverage of the urban area. This figure shows the Google Earth image with coloured lines showing tracks walked or cycled along streets in Jaisalmer during the survey.

A mid-point in time (end May 2009) of the TOLFA project was taken in order to calculate the number of surviving notched dogs at the time of survey. Applying the estimate of 0.70 for annual survival, 862 of the original 1000 sterilised dogs were estimated to be alive in the city at the time of surveying. 29% of the 523 dogs observed over seven separate survey tracks were ear-notched. This gives a point estimate of 2,962 roaming dogs in Jaisalmer in November 2009. Given that only 5 months had elapsed since the 1000 ear-notched dogs were released the CV for the estimate of 862 surviving is reduced to 0.024 and combined with a CV of 0.208 for the estimate of percentage ear-notched gives 95% confidence limits from 1,721 to 4,202 roaming dogs. Those limits are conservative because the CV calculated for the estimate of percentage ear-notched is likely to be an overestimate, being based on replicate survey tracks in non-overlapping areas of the city (Figure 5). That design was dictated by lack of time but means that individual replicates fail to integrate over the spatial variation in percentage ear-notched.

### Lactating and pregnant females

**Figure 6:**
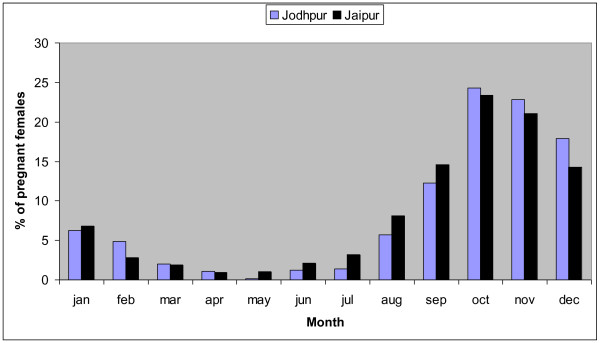
**Percentage of females found to be pregnant when spayed per month in Jodhpur and Jaipur**. - When female dogs are spayed they are sometimes found to be pregnant and the percentage of pregnant females changes over the year with seasonal breeding. This graph compares the percentage of the total females found to be pregnant when spayed that falls in each calendar month; data for Jaipur and Jodhpur reveal a close similarity in seasonal trend.

Clinical data collected during the ABC programmes in Jaipur and Jodhpur reveal a very similar strong seasonality in breeding. Figure 6 shows the percentage of total females found to be pregnant when spayed in each calendar month (the Jaipur data is also reported in [11] and an earlier dataset in [12]). Because of the seasonality in breeding, the population age structure and percentage of dogs ear-notched are also likely to vary seasonally.

During the November 2009 surveys, the percentage of non-spayed females that were visibly lactating was also calculated. Because of the similarity in breeding season we were able to compare those percentages. In Jodhpur the percentage of unspayed females that were lactating was, at 11.4%, significantly lower (P < 0.005) than the 26.1% in Jaipur and 32.5% in Jaisalmer.

## 4. Discussion

If, as is usually the case, the dogs released during an ABC programme are permanently marked those marks can be exploited to estimate and monitor the number of roaming dogs in the area covered by the programme. In comparison to marking dogs temporarily for a sight-resight experiment the obvious disadvantage of the marks applied during the ABC program is the need to estimate the number surviving till the time of the survey. The advantage is in the size of the marked sample and particularly its geographical extent. Previous surveys conducted in Cairo in 2005 and Colombo in 2007 (WSPA unpublished data) indicate extreme geographical variation in numbers of roaming dogs, the reasons for which are as yet not fully understood. Sight-resight experiments in limited areas are also vulnerable to unknown levels of mixing across the boundaries of the area that can bias the results. Furthermore, as compared to the other methods of estimating roaming dog populations (questionnaires, paint marking, distance methods and exhaustive counts) the use of the sample of dogs marked as part of the intervention is resource efficient. However, to exploit the existence of that marked sample it is essential that the numbers, dates and locations of all dogs released are reliably recorded from the start of the intervention.

Concerning the assumptions required for the method, ear-notches provide a permanent marking so the assumption of zero mark loss is justified. The marked dogs and the surveys designed to estimate their percentage in the population extend across the entire city so that immigration and emigration to and from the surveyed population are unlikely to be significant and the assumption of a closed population is also justified. However, the assumption that marked and unmarked dogs are equally likely to be caught or re-sighted may be violated. Dogs vary, by virtue of their behaviour and location, in the probability that they will be collected and may also vary in the probability that they will be observed later. For example, certain dogs maybe more likely to be both caught and resighted later than others. It may be possible to mitigate any such effect, for example by deliberately including in the surveys areas where dog catching is impractical but we have no data to assess the degree to which this assumption is violated. As a result, the estimate of roaming dog population size may be biased, however, unless the dog collection and survey methods are changed, any bias is likely to remain consistent and is therefore unlikely to preclude reliable monitoring.

Ear-notch marks are permanent but do not indicate when the mark was applied. HIS routinely tattoo the ear in addition ear-notching and those tattoo marks allowed annual survival of the released dogs to be estimated [11]. The tattoos are individually distinct and thus allow other parameters such as those related to movement to be estimated. It can however be difficult to read the detailed markings required and to estimate survival a mark giving the year of release would be sufficient. Given the importance of the survival parameter we suggest that tattoo marks giving at least the year of release should be applied routinely using a strict protocol to ensure longevity of the tattoo as part of any ABC programme. If finances allow the use of microchips would be ideal.

It is relevant to note, even when percentage ear-notched is estimated using specifically planned surveys rather than counts obtained opportunistically by the catching teams, that the survey efficiency is greatly enhanced if the survey includes at least one person normally employed as part of a catching team. The ability to distinguish most ear-notched from entire dogs quickly and reliably from a distance is an essential part of their normal work.

It is also important to note that seasonality in breeding, as reflected by the percentage of pregnant females at the time of spaying in Jodhpur and Jaipur (see Figure 6), will lead to seasonal differences in age structure. The percentage of pups in the population will vary seasonally and therefore so will the percentage of dogs ear-notched. Thus surveys used to monitor population size over time via the percentage of dogs ear-notched should be conducted at the same time in each year.

The data from the Pink City area of Jaipur (see Figure 4) suggest that the counts along the standard route detect about a third of the number of roaming dogs in the area. The counts and estimates both suggest a similar overall decline in numbers since 1998 (see [3]). The estimates suggest less change over the last five years, however estimates of reduction in the population based on percentage notched may be conservative for two potential reasons. As an ABC programme progresses the proportion of young dogs in the unsterilized population increases and hence the average age of the dogs sterilised is likely to decrease. Young dogs, particularly those below one year old, are likely to have a lower survival than dogs over one year old and hence the estimate for the number of surviving marked dogs may be overestimated. Also, if the percentage of notched dogs is estimated by catching teams as part of their normal catching routine the percentage may be biased downwards as these teams may focus on areas where fewer dogs have already been sterilised.

The main objective of this paper is to describe a resource efficient method of monitoring the number of roaming dogs; however the following observations may be of general interest.

The percentage of non-spayed females that were visibly lactating was found to be significantly lower in Jodhpur as compared to the other two cities. In Jodhpur, all male dogs collected are castrated as well as vaccinated and the percentage of castrated males seen during the November 2009 surveys was very high at 78.5%. In Jaisalmer the percentage castrated was 28.9%, in Jaipur 70% of males were ear-notched but according to the clinic records less than a third of ear-notched males are castrated. Although the fecund female population is normally assumed to be the limiting factor in population growth, these results suggest that a very high level of adult male castration may contribute to a reduced reproductive rate. We suggest this is a possibility worth further investigation.

We have no independent estimate of juvenile survival but it is likely to have increased with reduction in population size. Thus an adjustment to the ABC procedure to increase the percentage of females spayed may be required if the population is to be reduced further. Locating unspayed adult females during the breeding season (peak whelping was estimated to be November 23^rd ^in [11]) can be difficult if they are nursing a litter of puppies. However in Jaipur, prior to breeding the catching teams routinely collect females in heat by following groups of male dogs as they congregate around females. Another possibility may be to use local information to locate litters and collect the female and surviving pups before they disperse. Regular observations of 17 different litters over two breeding seasons in Jaipur have shown that the female and last surviving pups do not disperse from the whelping/rearing area till about 90 days after the whelping date.

## 5. Conclusions

The World Health Organisation recognises that data on the ecology of street dogs are limited and that data collection needs to be extended to areas for which none exist [1]. The mark-resight survey methodology described here is a simple way of providing population estimates for cities with current or recent ABC programmes that include visible marking of dogs; repeating such surveys on a regular basis will further allow for evaluation of ABC programme impact on population size and reproduction in the remaining unsterilised dog population.

## 6. Methods

The method used to estimate the number of roaming dogs was devised to be extremely simple in order to encourage impact assessment by those organisations and authorities responsible for implementing or evaluating ABC programmes. If the total number of roaming dogs is denoted by *R *and the number of surviving ear-notched dogs is denoted by *r*, then the expected percentage, *p*, of ear-notched dogs in a random sample of roaming dogs observed during a survey equals *100r/R*. Hence at any given time, a moment estimator for *R *will equal 1*00r/p*. The following sections consider estimation of *r *and *p*.

### The current size of the marked population, r

All dogs subject to sterilisation and/or vaccination are ear-notched using a clamp and cauterizer whilst under anaesthetic (see Figure 1). Given records of the number of ear-notched releases and an estimate of their annual survival it is straightforward to calculate the number of surviving ear-notched dogs. If *n_i _*ear-notched dogs are released in the *i^th ^*year after the start of the intervention the number surviving at the *y^th ^*year is where *S *is the annual survival of ear-notched dogs. If release numbers are available by month a more accurate calculation of the number surviving at *m *months after the start of the intervention is 

Very few estimates of survival are available for roaming dogs. Questionnaire surveys of dog owners in Dar es Salaam in 2006 and Colombo in 2007 provided annual survival estimates of 0.63 and 0.865 respectively for owned dogs that may be free to roam at certain times of day (WSPA unpublished data). However to estimate survival of all dogs caught and released back on to the streets, irrespective of whether or not they are owned, requires that a number are marked to give at least the year of their release and that a sample of such dogs is then available for inspection. Individually distinct tattoos applied by HIS to dogs released in Jaipur provided suitable data and yielded an estimate of 0.70 for annual survival of female dogs released there with 95% confidence limits from 0.62 to 0.78 [11], corresponding to a CV of 0.057.

Any error in the estimate of *S *will of course result in error in the estimated number of surviving ear-notched dogs. After a single year the estimated number of surviving ear-notched dogs is simply *n*_1_*S *and has therefore the same CV as the estimate of *S *but the CV increases later as higher powers of *S *are included. It does not, however, increase indefinitely. Using *E*(*S*) and *V*(*S*) to denote the expectation and variance of the survival estimate, the CV of the estimated number of surviving ear-notched dogs approaches an asymptotic value of  as the summation  tends to *n*/(1-*S*).

Substituting the expectation and variance of the Jaipur survival estimate for *E*(*S*) and *V*(*S*) gives a CV of at most 0.13 for any estimate of the number of surviving ear-notched dogs based on the Jaipur survival value.

### Surveys to estimate the percentage of ear-notched dogs, p

The current percentage of ear-notched dogs can be estimated using data collected during the dog catching work or by street surveys. Direct observation without the need to handle dogs is sufficient to establish the mark status (ear-notched or not), age, sex and reproductive status (pup, adult lactating female, adult non-lactating female or adult male) of each dog seen. Pups are defined as those below approximately 4 months of age. Lactating females are those with visibly swollen mammary glands, i.e. not only large teats, as can occur in females that have had previous litters, but those visibly carrying milk and therefore assumed to be currently feeding a litter of pups.

The street survey designs attempted to avoid over or underestimating the percentage of ear-notched dogs over the whole city. In survey of wildlife populations such biases can be avoided by randomising the location of the survey effort, using for example a randomly positioned grid of survey lines, to give each animal an equal probability of inclusion in the sample. That ideal can not be achieved in an urban environment where many locations can not be accessed and the best possible alternative was to cover as much ground as possible and as evenly as possible. A standard procedure was adopted with respect to distance searched to either side of the street and time taken to search areas of open ground.

We assigned each dog counted to one of the city zones used to record the monthly releases of sterilised and vaccinated dogs. This provided the potential to estimate roaming dog numbers by city zone and hence avoided geographical variation in percentage ear-notched biasing the estimate for the city as a whole.

#### Jaipur surveys

In Jaipur the vehicles used by the catching teams are fitted with event counters to record the number of ear-notched and entire male and female dogs seen while the teams are in transit to the catching area or searching for dogs. In addition HIS have for many years conducted street counts of roaming dogs in the Pink City area. Two counts are completed each year by walking a standard route. Thus it was possible to calculate a sequence of population estimates for the Pink City area based on percentage ear-notched seen in that area during the street counts and compare them to the counts themselves.

#### Jodhpur surveys

In Jodhpur the number of ear-notched and entire male and female dogs seen while the teams are in transit to the catching area or searching for dogs is recorded by the supervisor accompanying each catching team. During November 2009, additional street surveys were conducted using bicycles and motorcycles to cover central areas of the city known to have received the most intensive intervention and peripheral areas where intervention effort had been less intensive. Those counts provided an estimate of the percentage of ear-notched dogs over the whole city, which was compared to the estimate based on data collected by the supervisors.

#### Jaisalmer surveys

In the same month, street surveys were conducted in Jaisalmer, only 5 months after the completion of the 1000 operations carried out in that city by TOLFA. As detailed street maps were unobtainable, a GPS receiver was used in combination with an aerial map downloaded from the Google Earth website (http://earth.google.co.uk/) to a hand-held microcomputer running the GPS*GO *mapping program under Windows 6 (Craporola Software Inc). A track was marked on the map as the survey team walked or cycled along the streets so a roughly even coverage of the urban area could be achieved (Figure 5).

### Calculating the CV of the roaming dog population estimates

We estimated the CV of the percentage of dogs ear-notched, *p*, using replicate survey tracks and used the delta method to combine the CV estimates for *r *and *p *to estimate the CV of the roaming dog population estimate:

## 7. Authors' contributions

JR, RW, RJ and BS ensured accurate recording of data relating to release of mark dogs. JR, EH and LH conducted the surveys in November 2009, this data was used in comparisons of percentage of lactating females between cities, for the population estimate in Jaisalmer and contributed to the population estimates for Jodhpur and Jaipur. LH and EH carried out analysis of data, provided interpretation of results and drafted the manuscript. JR provided interpretation of analysis results. All authors read and approved the final manuscript.
